# Emerging Human Babesiosis with “Ground Zero” in North America

**DOI:** 10.3390/microorganisms9020440

**Published:** 2021-02-20

**Authors:** Yi Yang, Jevan Christie, Liza Köster, Aifang Du, Chaoqun Yao

**Affiliations:** 1 Department of Veterinary Medicine, College of Animal Sciences, Zhejiang Provincial Key Laboratory of Preventive Veterinary Medicine, Zhejiang University, Hangzhou 310058, China; yangyi0607@zju.edu.cn; 2 The Animal Hospital, Murdoch University, 90 South Street, Murdoch, WA 6150, Australia; jevanchristie@gmail.com; 3 Department of Small Animal Clinical Sciences, College of Veterinary Medicine, University of Tennessee, 2407 River Drive, Knoxville, TN 37996, USA; lkoster@utk.edu; 4 Department of Biomedical Sciences and One Health Center for Zoonoses and Tropical Veterinary Medicine, Ross University School of Veterinary Medicine, Basseterre 00334, Saint Kitts and Nevis

**Keywords:** human babesiosis, *Babesia* spp., *Babesia microti*, *Babesia divergens*, *Babesia venatorum*, *Babesia duncani*, *Babesia crassa*

## Abstract

The first case of human babesiosis was reported in the literature in 1957. The clinical disease has sporadically occurred as rare case reports in North America and Europe in the subsequent decades. Since the new millennium, especially in the last decade, many more cases have apparently appeared not only in these regions but also in Asia, South America, and Africa. More than 20,000 cases of human babesiosis have been reported in North America alone. In several cross-sectional surveys, exposure to *Babesia* spp. has been demonstrated within urban and rural human populations with clinical babesiosis reported in both immunocompromised and immunocompetent humans. This review serves to highlight the widespread distribution of these tick-borne pathogens in humans, their tick vectors in readily accessible environments such as parks and recreational areas, and their phylogenetic relationships.

## 1. Introduction

*Babesia* spp. are piroplasm parasites of various vertebrate animals with host specificity. Their infections may cause clinical manifestations such as fever, anemia, or even death although asymptomatic infections are not unusual. Some *Babesia* spp. are zoonotic, causing human infections that result in babesiosis. The very first case of human babesiosis appeared in the literature in 1957. This fatal disease was diagnosed in a 33-year-old asplenic man from Zagreb [1]. The disease was so rare back then that by 1968 only three human cases were reported [2]. Nevertheless, human babesiosis has been emerging in recent years in many geographical regions around the world, particularly in the United States of America (USA), Canada, and China. In the last decade, reviews have been published on the *Babesia* spp. life cycle, pathogenesis, immunity, diagnosis, and treatment as well as human babesiosis in Europe and China [3,4,5,6]. We reviewed in this manuscript; (a) human babesiosis cases that had been diagnosed as *Babesia* species by molecular confirmation with attention to cross-sectional surveys, (b) vectors for these *Babesia* spp., (c) *Babesia* spp. in tick vectors collected in the recreational areas readily accessible to humans and possible roles by the domestic dog in human babesiosis. We further performed a phylogenetic analysis of the *Babesia* spp. in human cases and those harbored by ticks recovered in the areas easily accessible to humans. We aimed to initiate a debate on this emerging disease and call the attention of both the medical and veterinary professionals.

## 2. Human Babesiosis

### 2.1. Challenge in Diagnosis of Babesia spp. in Humans

Individual *Babesia* spp. usually have a rather narrow spectrum of hosts, i.e., each with strict host specificity. For instance, *Babesia canis* causes canine babesiosis in domestic dog (*Canis lupus familiaris*). So far, no *Babesia* spp. are found exclusively using only humans as a host although several species have been found infecting humans. Due to technical challenges in the diagnosis of *Babesia* spp. in humans that have similar morphology and cross-reaction of antibody and antigen, molecular techniques such as PCR and DNA sequencing are often required for species identification. So far, *Babesia* spp. that have been confirmed infecting humans by molecular methods include *B. microti*, *B. divergens*, *B. venatorum* (Babesia sp., EU1), *B. duncani* (Babesia sp., WA1), *B. crassa*, and two yet to be named species *Babesia* sp. KO1 and *Babesia* sp. CN1 (*Babesia* sp. XXB/HangZhou).

### 2.2. Geographical Distribution

**Figure 1:**
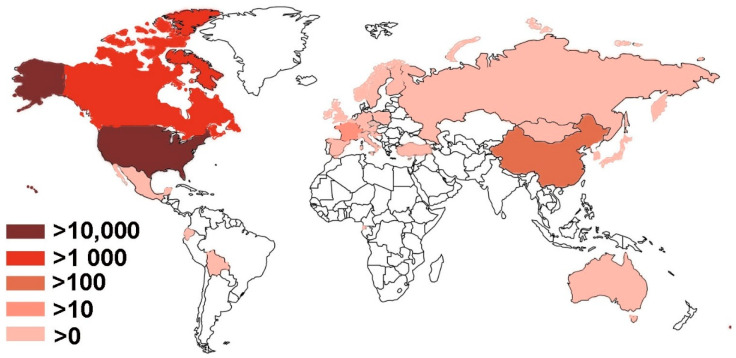
Geographical distribution of human babesiosis. The darker the color, the more numerous cases there are. There are no reported cases in unfilled countries or regions.

Human babesiosis has been reported in 28 countries on all continents except Antarctica (Table 1 and Figure 1). The data were collected from MEDLINE by searching PubMed using *Babesia*, OR babesiosis, OR babesiasis AND human case. The resultant titles and abstracts were browsed to identity papers with human cases. The references of those papers were also monitored for additional relevant publications. All countries except the USA, Canada, China, and France, have fewer than ten cases reported and will be discussed in less detail. On the other hand, the USA and Canada, two countries in North America, account for more than 95% of worldwide reported cases and therefore are “the ground zero” of this current emergence of human babesiosis that is mainly attributed to *B*. *microti*. Human babesiosis has been a USA notifiable disease nationally since January 2011 (https://www.cdc.gov/parasites/babesiosis/index.html (accessed on 17 February 2021)). The total annual cases reported to CDC are 14,042, i.e., 1126 cases for 2011, 911 for 2012, 1761 for 2013, 1742 for 2014, 2074 for 2015, 1909 for 2016, 2358 for 2017 and 2161 for 2018 (https://www.cdc.gov/parasites/babesiosis/data-statistics/index.html (accessed on 17 February 2021)). No data are available for 2019 yet. Furthermore, a retrospective population-based study was carried out among elderly, over 65 years old, Medicare beneficiaries between 2006 and 2013 using the Centers for Medicare & Medicaid Services (CMS) databases. A total of 10,305 cases had a recorded diagnosis of babesiosis. The top five states with the highest average rate per 100,000 residents annually included Connecticut 46 (total case 1307), Massachusetts 45 (2161), Rhode Island 42 (247), New York 27 (3193), and New Jersey 14 (980). A seasonal peak occurred in the summer between May and October accounting for approximately 75% of all reported cases [7]. It is likely some cases covered in CMS for the years 2011–2013 of 1366, 1219, and 1848 cases, respectively, may be also reported to CDC. The maximum of babesiosis cases in the USA between 2006 and 2018 can be calculated provided that no cases overlap between CDC and CMS data, which were 24,347. The minimal numbers were 20,549 if all CDC cases were included in CMS for the years 2011–2013.

*Babesia duncani*, the former *Babesia* sp., WA1, was a new species found in humans in the Western USA, predominantly in Washington and California. So far, a total of 14 human babesiosis cases caused by *B. duncani* have been reported in the USA, all of them in western states [43]. In addition to *B. microti* and *B. duncani*, *B. divergens* has been reported in Kentucky, Missouri, Michigan, and Washington (Table 1).

The country with the 2nd most numerous reported cases of human babesiosis is Canada where *B*. *microti* and *B*. *duncani* have been confirmed [39,40]. From 2011 to 2017 data on *B. duncani* infections in Canadians were collected by tick-borne disease specialists (10 physicians and 10 naturopathic physicians) in the USA and Canada. Cases were found in all ten Canadian provinces ranging from as few as four within Newfoundland and Labrador, seven within Prince Edward Island to as many as 365 within Ontario, and 377 within British Columbia. There was a steady increase in the number of infections annually during the collection period of seven years, ranging from 119 in 2011 to 198 in 2017. In total 1,119 cases were identified [41]. Nevertheless, it is worthy of pointing out that testing in this paper was done at five US laboratories that used in-house assays of *B. duncani* WA1 IgG by the immunofluorescence assay (IFA) and the DNA probe of the internal transcribed spacer of rRNA. There was no breakdown of which tests were positive and how many samples at each lab. Therefore, more evidence from Public Health laboratories or other researchers is warranted to corroborate these numbers.

China has the 3rd largest number of reported cases of human babesiosis. China is the world’s most populous country, approximately 2.5 times the population of North America, which adds credence to the significance of the reported prevalence in North America. Sixteen cases of human babesiosis were diagnosed with *B. microti* infections (Table 1). The first Chinese case of *B*. *venatorum* infection was from an eight-year-old boy with the diagnosis made based on microscopy, PCR, and DNA sequencing [9]. Additional 48 cases were confirmed. All cases were from immunocompetent individuals. Thirty-two patients had clinical manifestations including fever, headache, fatigue, and dizziness [10]. A survey was carried out from May to July in the Heilongjiang Province of China in 2015 and 2016 among the patients who presented to a local hospital with flu-like symptoms and a tick-bite history. Fifty-eight of the 1125 patients (5.2%) were confirmed to have *B. crassa* DNA in their whole blood through species-specific PCR and DNA sequencing [11]. Further, a Chinese patient suffering from multiple episodes of mild fever and fatigue of unknown origin over a period of 10 years was diagnosed with babesiosis through microscopy, PCR, and DNA sequencing. The parasite was given a new species name as *Babesia* sp. XXB/HangZhou since its 18S rRNA sequence was different from, although being clustered with *B. microti* and *B. duncani* by phylogenetic analysis [12]. Nevertheless, the new name is inconsistent with the International Code of Zoological Nomenclature (http://iczn.org/iczn/index.jsp (accessed on 17 February 2021)) or in the tradition of *Babesia* naming. Thus, the name of *Babesia* sp. CN1 would probably fit better until a valid name is acquired.

### 2.3. Cross-Sectional Survey of Babesia spp. Infections in Humans

Although human babesiosis has emerged as an important disease and has been notifiable in the USA since 2011, its prevalence among humans has been scarcely addressed. The following cross-sectional data may provide a glimpse.

In a serological survey to detect IgG antibodies against *B. microti* using IFA, four were positive out of 879 blood donors (0.5%) from Oregon, California, Arizona, North Dakota, Texas, Kansas, Alabama, Maryland and Massachusetts during 2009 [44]. A New Jersey medical center performed multiplex quantitative polymerase chain reaction (qPCR) to detect both *B. microti* protozoan and *Borrelia burgdorferi* bacterium in blood samples collected from the residents of three New Jersey counties, an endemic area for both pathogens. Among 192 samples 12 (6.3%) were positive for *B. microti* alone and 36 (18.7%) had co-infection of both *B. microti* and *B. burgdorferi* [45]. In a cross-sectional study in Switzerland from December 1997 to May 1998, blood samples were collected and tested for IgG antibodies to *B. microti*. Five of 396 (1.3%) were found positive [20]. In a serological survey carried out in Sweden from 2014 to 2015 using IFA, 10.5% (9/86) and 1.2% (1/86) of *B. burgdorferi* s.l. antibody positive individuals had IgG and IgM antibodies to *B. microti*, respectively. In contrast in the healthy controls, the rate for the corresponding antibodies was 1.5% (3/197) and 0.5% (1/197), respectively [37]. In 2013 a cross-sectional survey of 271 healthy individuals in two rural communities in Bolivia using microscopy, PCR and DNA sequencing confirmed nine (3.3%) cases having a *B. microti* infection [50]. A similar cross-sectional study in Mongolia surveyed 100 farmers in 2011, 7% (7/100) and 3.0% (3/100) were found to be positive using IFA and molecular methods including PCR and DNA sequencing, respectively [19]. In a serological survey carried out in Sweden as having been described earlier, 7.0% (6/86) of *B. burgdorferi* s.l. antibody positive individuals were also IgG positive to *B. divergens*. In contrast in the healthy controls, the rate for IgG and IgM antibodies were 1.0% (3/197) and 0.5% (1/197), respectively [37]. In a survey of clinical sera collected from the USA pacific regions (Oregon, California) and other states (Arizona, North Dakota, Texas, Kansas, Alabama, Maryland, Massachusetts) during 2008 and 2009 by IFA, 27.0% sera were IgG positive for *B. duncani*. Furthermore, 18 out of 879 blood donors (2.0%) from these states were IgG positive for the same parasite [44] (Table 1). Collectively, humans appear to be more frequently exposed to *Babesia* spp. infection than clinically diagnosed cases have indicated in many geographical locations. Therefore, babesiosis should be included in differentials of peoples with fever, especially those with a tick-bite history.

## 3. Vectors

All *Babesia* spp. require a hard tick vector in their life cycle as a definitive host. Various hard tick vectors of *Babesia* spp. infest humans (Table 2). A prospective study performed in the Netherlands between January 2004 and December 2008 examined ticks that had fed on human beings, and individuals were followed for a minimum of 6 months with their clinical outcomes documented. All ticks were *Ixodes ricinus* and they were tested for tick-borne pathogens including *Babesia* spp. using PCR and reverse line blotting techniques. 9.1% and 0.3% out of 297 ticks were positive for *B. microti* and an undetermined *Babesia* sp., respectively. Clinical symptoms were associated with a tick attachment duration lasting greater than 24 h [52]. It was also found that 14 of the 64 pools of 408 *I. ricinus* ticks collected from Switzerland tested positive for *B. microti* by PCR and DNA sequencing [20].

High throughout real-time PCR was carried out to screen 7050 *I. ricinus* nymphs collected within France in 2011, Denmark in 2012 and the Netherlands between 2008 and 2012. Primers and probes were designed for real-time TaqMan PCRs to target various genes of several *Babesia* spp. including *B. microti, B. divergens, B. caballi, B. canis, B. vogeli, B. venatorum, B. bovis, B. bigemina, B. major*, and *B. ovis*. Confirmation was performed by conventional PCR in the amplification of 18S rRNA gene followed by DNA sequencing. *Bebesia divergens* was found only in ticks collected in Denmark and the Netherlands while *B. venatorum* was found in all three countries [53]. *Ioxdes ricinus* ticks collected from Norway between 2006 and 2008 were individually tested for *Babesia* spp. by PCR and DNA sequencing. A total of 17 out of 1908 (0.9%) ticks were found to be positive for *Babesia* spp. with *B. divergens* accounting for less than 30% of the *Babesia* spp. identified and *B. venatorum* accounting for 71% of the positive samples [54].

A number of studies have examined *B. venatorum* transmission in potential vectors. In an experiment of in vitro transmission of the parasite by *I. ricinus*, nymphs and adult females of field-collected ticks that fed on a glass feeder until fully engorged. The feeder contained 3 mL of sheep red-blood-cells (RBC) with approximately 8% parasitemia. Parasite DNA was detected from eggs and larvae derived from the infected adult females. Furthermore, infected adult females transmitted the *Babesia* spp. during a new blood meal with parasite DNA being detected in their salivary gland [55]. *Ixodes ricinus* ticks in Switzerland were found to be 0.8% positive for *B. venatorum* by next generation sequencing [56]. Ticks were collected from humans in Italy between 1995 and 2011. Among the 334 *I. ricinus*, 0.3% (one) was positive for *B. venatorum* [57]. *Ixodes ricinus* adults were collected from cattle farms (223 ticks) and wild fauna reserve (31 ticks) after full engorgement, 1.3% (three) and 6.5% (two) adult ticks were positive for sporozoites in their salivary gland, respectively. These sporozoites further infected only sheep RBCs [58]. In the Czech Republic, *I. ricinus* ticks were collected and tested for *Babesia* spp. by amplifying a fragment of the 18S rRNA gene. *Babesia venatorum* was confirmed from two positive amplicons [59]. *Ixodes persulcatus* (819) and *Haemaphysalis concinna* (260) ticks in China were tested by PCR and DNA sequencing for *Babesia* spp. Only *I. persulcatus* was found to be positive (1.0%, 8/819) and DNA sequencing confirmed it as *B. venatorum* [9,10]. *Ixodes persulcatus* ticks in Mongolia were also found to be positive for *B. venatorum* in two studies, one found a prevalence of 3.2% (2/63) [60] and the other found a prevalence of 3.3% (9/275) [61].

The vector and reservoir of *B. duncani* had been elusive for over two decades since the parasite was first discovered in a patient in Washington State, USA. Lately, DNA of *B. duncani* was found in the larval and adult stages of the winter tick, *Dermacentor albipictus*. Overall, a minimum infection prevalence in larvae was 7.2% with the highest rate of 20.7%, whereas rate in adults was 2.1% [43]. The same study also confirmed a primary reservoir was mule deer (*Odocoileus henionus*) [43].

Ticks were collected from May to July in 2014 in the Heilongjiang Province of China and were analyzed through species-specific PCR and DNA sequencing for *B. crassa* DNA. Eight of the 1296 (0.6%) *I. persulcatus* ticks and one of 252 (0.4%) *H. concinna* ticks were found to be positive [11].

Collectively, *I. ricinus* has been confirmed a vector for *B. microti*, *B. venatorum* and *B. divergens* in Europe. *Ixodes persulcatus* is a vector for *B. venatorum* and *B. crassa* in Asia. *Haemaphysalis concinna* is a vector for *B. crassa* in Asia. *Dermacenter albipictus* is the vector for *B. duncani* in North America.

## 4. Tick Vectors Found in the Recreational Areas Readily Accessible to Humans

Ticks were collected all-year-round in 2011 in the Insugherata Natural Reserve located in northwestern Rome. The ticks that were PCR positive for the 18S rRNA gene of *B. microti* were *Rhipicephalus turanicus,* 1.2% (1/85, 29 males and 56 females) and *I. ricinus*, 12.1% (4/33, 11 males and 22 females) while *D. marginatus* (1 male and 6 females) and *H. punctate*, (1 male and 3 females) were found to be negative [62]. Three parks in the Emilia-Romagna Region of Northern Italy were surveyed for ticks from April to October 2010 every 15 days. Picnic areas and footpaths that were frequented by people were selected. DNA was extracted from individual adult ticks or pools of 5 nymphs or 10 larvae from the same area at the same time point followed by PCR and DNA sequencing of piroplasm’s 18S rRNA. In total, 6.4% of the male ticks (2/31), 4.8% of the female ticks (1/21), 9.9% of the nymph tick pools (25/232), and 0.0% of the larvae (0/32) were PCR positive. Eleven including nine pools of nymphs and two adults were positive with *B. venatorum*. Two pools of nymphs were positive with *B. divergens* and *B. capreoli* [63]. Ticks were collected in the Tri-City Landscape Park in northern Poland during 2009–2010. The park was a destination for tourism and leisure among residents of the cities of Gdańsk, Sopot, and Gdynia. 4.5% (34/757) of the *I. ricinus* ticks were found to be *Babesia* sp. positive through PCR with *B. venatorum* being the predominant species found [64]. In a survey in Bratislava, Slovakia, a total of 2799 *I. ricinus* ticks were collected and this represented an urban/suburban habitat and was characterized by significant human development between 2011 and 2013. Thirty-three ticks (1.2%) were positive for *Babesia* spp. which included *B. microti*, *B. venatorum*, and *B. divergens* [65]. Ticks were also collected between April and June 2008 from a suburban forest, Sénart Forest, in the southern Paris metropolitan area. This forest has three million visitors annually. Five hundred and fifty-eight out of the 574 ticks identified were *I. ricinus* ticks. *Babesia* sp. was tested by PCR and DNA sequencing and among these *I. ricinus* ticks, an estimated overall prevalence of 1.6% was established. All parasites were identified by DNA sequencing as *B. venatorum* [66].

In short, ticks collected in areas with high human activities such as parks and picnic areas include confirmed vectors for human babesiosis such as *I. ricinus*. Further, PCR and DNA sequencing have detected the DNA of many *Babesia* spp. including *B. microti*, *B. venatorum*, and *B. divergens* in these ticks.

## 5. Phylogenetic Analysis of *Babesia* spp. Harbored by Humans, and Ticks on These Hosts

**Figure 2:**
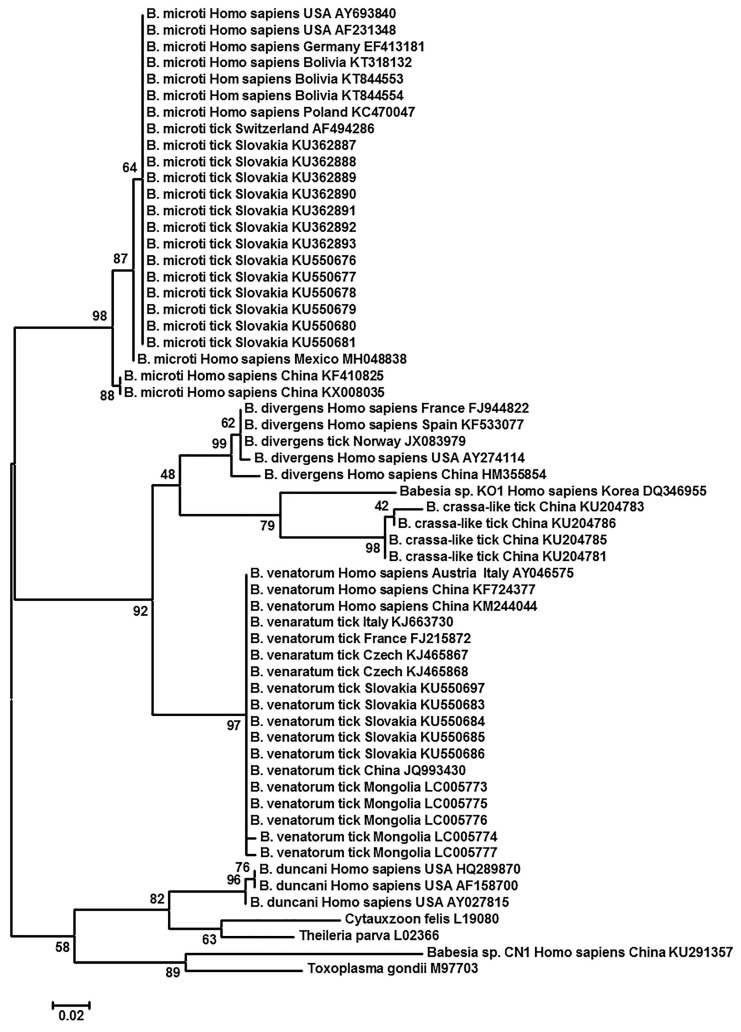
Phylogenetic analysis of 18S rRNA sequences of *Babesia* spp. found in human and tick vectors. The Maximum Likelihood method was performed in a default setting of the Jukes–Cantor model, uniform rates, and complete depletion with 1000 bootstrap replications. The trees were rooted with *Toxoplasma gondii*. Two species of piroplasms, one each in the closely related genera of *Cytauxzoon* (*C. felis*) and *Theileria* (*T*. *parva*) were also included. Scale bar indicates nucleotide substitutions per site. Numbers at the horizontal lines represent the percentage of replicates of 1000 repeats. The Neighbor-jointing method in the default setting yielded almost identical results as Maximum Likelihood did (Not shown). Each entry was identified in the order of *Babesia* sp., host, geographical region, and Accession number.

To understand the relationship between *Babesia* spp. that had been identified from humans, and tick vectors on humans in different geographic regions a phylogenetic analysis was carried out using 18S rRNA sequences of *Babesia* spp. The trees were rooted with *Toxoplasma gondii*. Two piroplasm species, one each in the closely related genera of *Cytauxzoon* (*C. felis*) and *Theileria* (*T*. *parva*) were also included. The three piroplasm genera are distinguishable from one another by morphological, ultrastructural, and parasite life-cycle characteristics like host preference and host cells of infection [67]. Both Maximum Likelihood (ML) and Neighbor-Joining (NJ) were performed with 1000 bootstrap replications using the free software MEGA (Version 5.2.2) [68]. Both ML and NJ yielded almost identical results. Only the ML result is shown in Figure 2. In general, each of *B. venatorum*, *B. crassa*, *B. divergens*, *B. microti*, and *B. duncani* forms its own clade with multiple isolates. It seemed that both *Babesia* sp. KO1 and *Babesia* sp. CN1 differed from one another and from other *Babesia* spp. They may each represent a new *Babesia* species (Figure 2). *Babesia* sp. KO1 was most closely related to *B. crassa*. Interestingly, *Babesia* sp. CN1 appeared to be closer to *T. gondii* than to two piroplasms, i.e., *C. felis* and *T. parva* (Figure 2). Likely, this may not be a *Babesia* sp. at all.

## 6. Possible Roles of the Domestic Dog in Human Babesiosis

May it even be possible that pet dogs play a role in human babesiosis? Firstly, dogs in China and Russia were found to be infected with *B. microti* by PCR and DNA sequencing [69,70]. Secondly, ticks collected from pet dogs carry *B. microti*, *B. venatorum*, and *B. divergens* [71,72,73,74,75,76,77,78,79]. These ticks might have obtained *Babesia* spp. pathogens from other hosts prior to their attachment to the pet dogs. Nevertheless, these pet dogs bring these *Babesia* infected ticks to the human households, making tick infestation and *Babesia* spp. transmission to humans a much higher possibility. Lastly, pet dogs living within the same household were incriminated for at least two cases of human babesiosis in the literature [80,81]. One needs to be cautious in interpreting this finding as no evidence presented linked those dogs directly to human infections. Collectively, there is no unequivocal evidence so far in the literature suggesting dogs contribute to human babesiosis. They may play a marginal role. Further experimental data are required to ascertain their roles.

## 7. Conclusions

This review aimed to address the geographic distribution of the human-infecting *Babesia* spp., their phylogenetic relationship, and their tick vector worldwide. Human babesiosis is caused by several *Babesia* spp. that includes but are not limited to, *B. microti*, *B. divergens*, *B. venatorum*, *B. duncani*, *B. crassa*, and two *Babesia* spp. strains i.e., *Babesia* sp. KO1 and *Babesia* sp. CN1. The latter two may represent a new *Babesia* species, which as of yet needs to be further defined and properly named.

The number of human cases that appeared in the literature has been exponentially increased in the last decade. Two countries in North America, USA, and Canada have over twenty thousand and one thousand cases, respectively. China is in distant third place with over one hundred cases. Cases have been reported from all continents with human residence. Several factors may contribute to this rapid increase in confirmed cases of human babesiosis. First is the awareness of the disease in medical professionals, resulting in the correct diagnosis of the disease which would have been mistakenly diagnosed as other infections. The second is through active monitoring and survey studies. Babesiosis is a notifiable disease in the USA. Cross-sectional surveys have been carried out in many regions including Canada and China. Third is global warming, which has expanded tick vector habitats to areas that can be readily accessed by humans such as parks and recreational areas. The fourth factor is transfusion transmission. From 2009 to 2016 in Massachusetts alone 45 of 2578 (1.7%) were transmitted by transfusion [82]. The last, but not least, factor is vertical transmission from an infected mother to her offspring [83].

## Figures and Tables

**Table 1:** Number of cases and prevalence of human babesiosis worldwide.

Countries	Number of Case *	Cross-Sectional Survey ^†^	References
**Africa**			
Equatorial Guinea	1 (*Bm*)		[8]
**Asia**			
China	16 (*Bm*); 1 (*Bd*); 49 (*Bv*); 1 (CN1); 58 (*Bc*)		[5,9,10,11,12,13,14,15,16]
Korea	1 (KO1)		[17]
Japan	1 (*Bm*)		[18]
Mongolia		7.0% (7/100) ^1^; 3.0% (3/100) ^2^	[19]
**Australia**			
Australia	1 (*Bm*); 1 (*Bdu*)		[20,21]
**Europe**			
Austria	1 (*Bm*); 2 (*Bv*)		[22,23,24]
Belgium	1 (*Bm*)		[25]
British Isles	6 (*Bd*)		[25]
Czech	1 (*Bm*)		[26]
Croatia	1 (others)		[25]
Finland	1 (*Bd*)		[27]
France	11 (*Bd*), 2 (others)		[25,28]
Germany	1 (*Bm*); 1 (*Bv*)		[29,30]
Italy	1 (*Bv*)		[23]
Norway	1 (*Bd*)		[31]
Poland	1 (*Bm*)		[25]
Russia	1 (*Bd*)		[32]
Slovenia	1 (*Bc*)		[33]
Spain	2 (*Bd*); 1 (*Bm*); 2 (others)		[25,34,35,36]
Sweden	1 (*Bd*)	2.0% (4/197) ^1^	[25,37]
Switzerland	1 (*Bd*)	1.3% (5/396) ^1^	[20,25]
Turkey	2 (*Bd*)		[38]
**North America**			
Canada	1,120 (*Bdu*); 1 (*Bm*)		[39,40,41]
Mexico	4 (*Bm*)		[42]
USA	24,363 (*Bm*); 14 (*Bdu*); 4 (*Bd*)	0.5% (4/879) ^1^; 2.0% (18/879) ^1^; 25.0% (48/192) ^2^	[7,43,44,45,46,47,48,49]
**South America**			
Bolivia		3.3% (9/271) ^2^	[50]
Ecuador	1 (*Bm*)		[51]

*: Abbreviations of *Babesia* spp. are *B. microti*—*Bm*; *B. divergens*—*Bd*; *B*. *venatorum*—*Bv*; *B*. *duncani*—*Bdu*; *Babesia* sp. KO1-KO1; *Babesia* sp. CN1-CN1; *Bc*—*B*. *crassa*. ^†^: ^1^: serology; ^2^: PCR.

**Table 2:** *Babesia* species causing human babesiosis, their vectors and geographical distribution.

*Babesia* spp.	Distribution	Confirmed Tick Vector	Possible Tick Vector
*B. microti*	North America	*I. scapularis*	
	Europe	*I. ricinus*	*H. concinna*, *R. turanicus*
	China	*I. persulcatus*	
	Equatorial Guinea	u	
	Bolivia	u	
	Ecuador	u	
	Japan	u	
	Mongolia	u	
*B. divergens*	Europe	*I. ricinus*	
	China	u	
	USA	u	
*B. venatorum* (*Babesia* sp., EU1)	Europe	*I. ricinus*	*I. canisuga*
	China and Mongolia	*I. persulcatus*; *I. ovatus*	
	Japan	u	
*B. duncani* (WA1)	North America	*Dermacentor albipictus*	*I. scapularis*
	Australia	u	
*Babesia crassa*	China	*I. persulcatus*, *H. concinna*	
*Babesia* sp. CN1	China	u	
*Babesia* sp. KO1	Korea	u	

u: unknown.

## Data Availability

All data generated or analyzed during this study are included in this published article.
